# Supplementary material for: Transcriptomic Responses of Salvia hispanica to the Infestation of Red Spider Mites (Tetranychus neocaledonicus)
Source: Int J Mol Sci. 2023 Jul 31;24(15):12261. doi: 10.3390/ijms241512261 (PMC10418447; doi:10.3390/ijms241512261)
Supplement: Supplementary file 1 [file ijms-24-12261-s001.zip › ijms-2507279-supplementary.pdf]

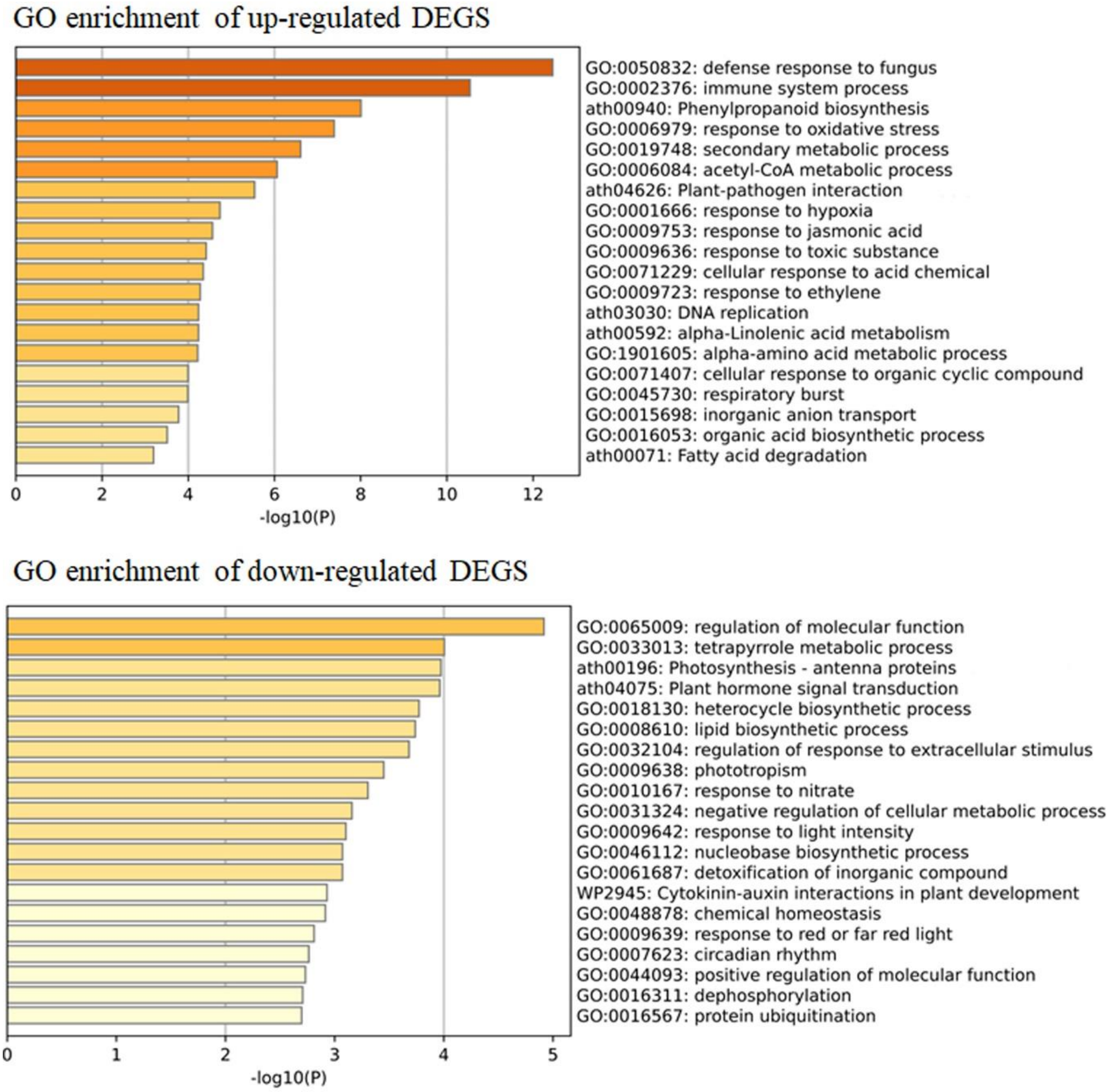

Figure S1 Gene ontology (GO) analysis of differentially expressed genes (DEGs) that are up-regulated and down-regulated between mite-feeding and controls of chia leaf transcriptomes at the significance level of $P < 0.05$.

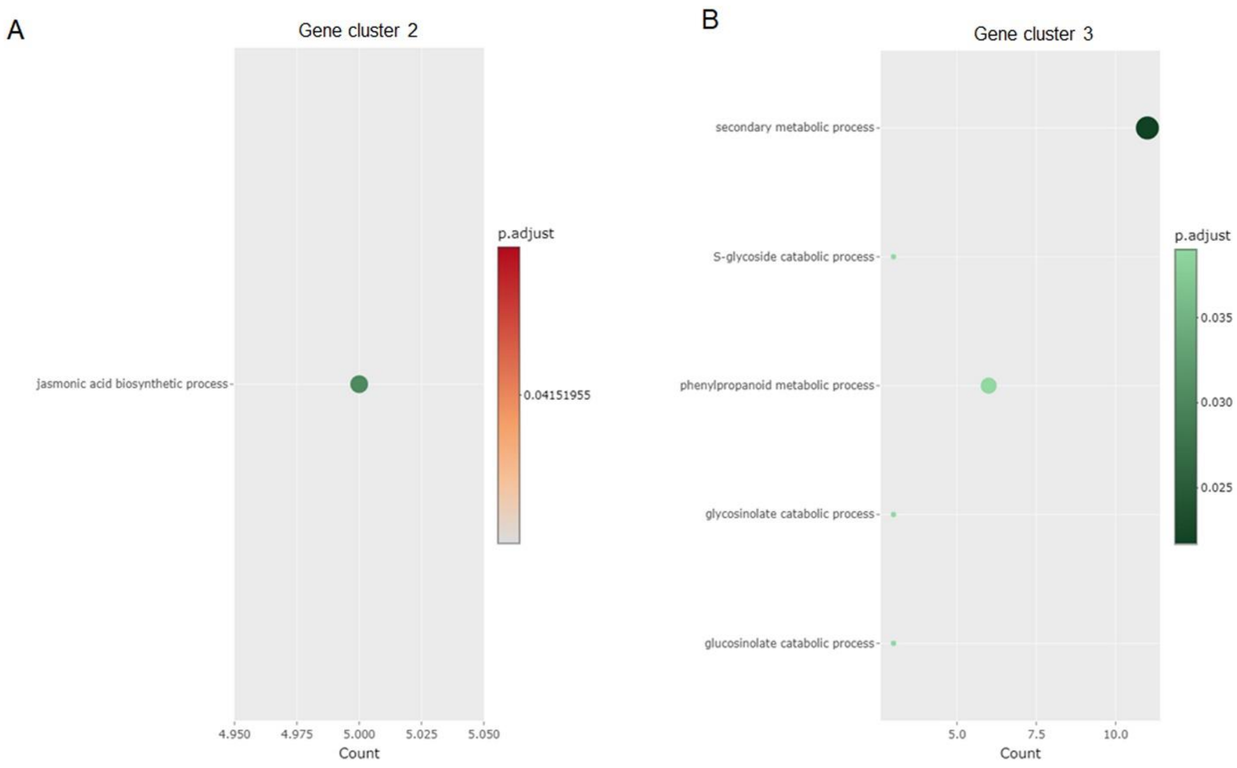

Figure S2 Gene ontology (GO) analysis of differentially expressed genes (DEGs) in gene expression cluster 2 (A) and 3 (B). GO terms at the significance level of 0.05 are indicated.

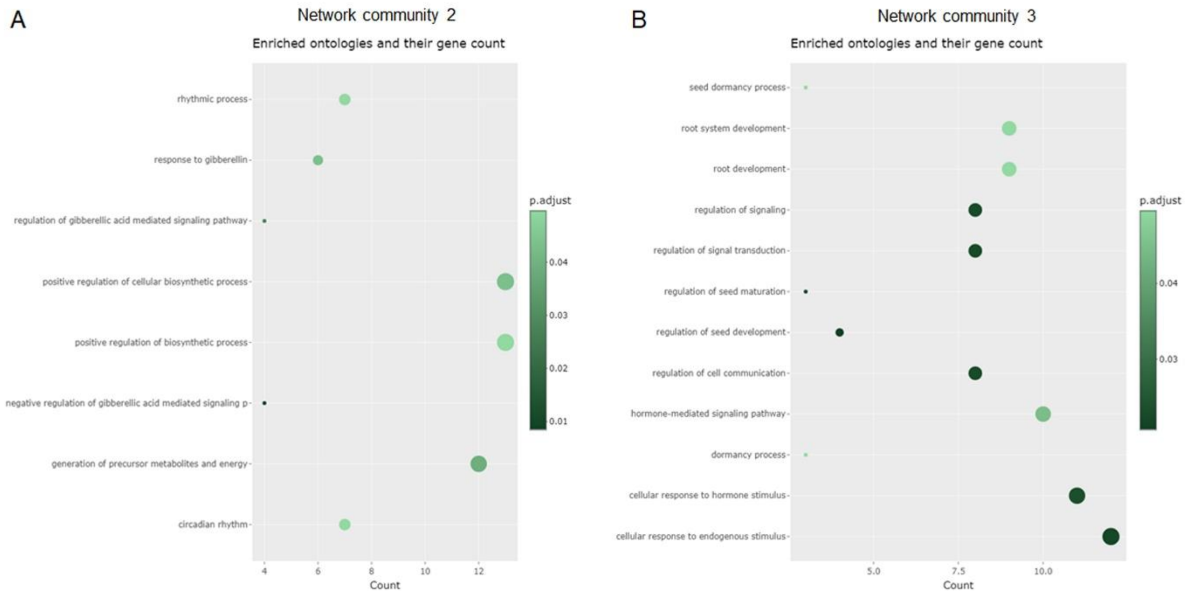

Figure S3 Gene ontology (GO) analysis of differentially expressed genes (DEGs) in gene regulation network community 2 (A) and 3 (B). GO terms at the significance level of 0.05 are indicated.

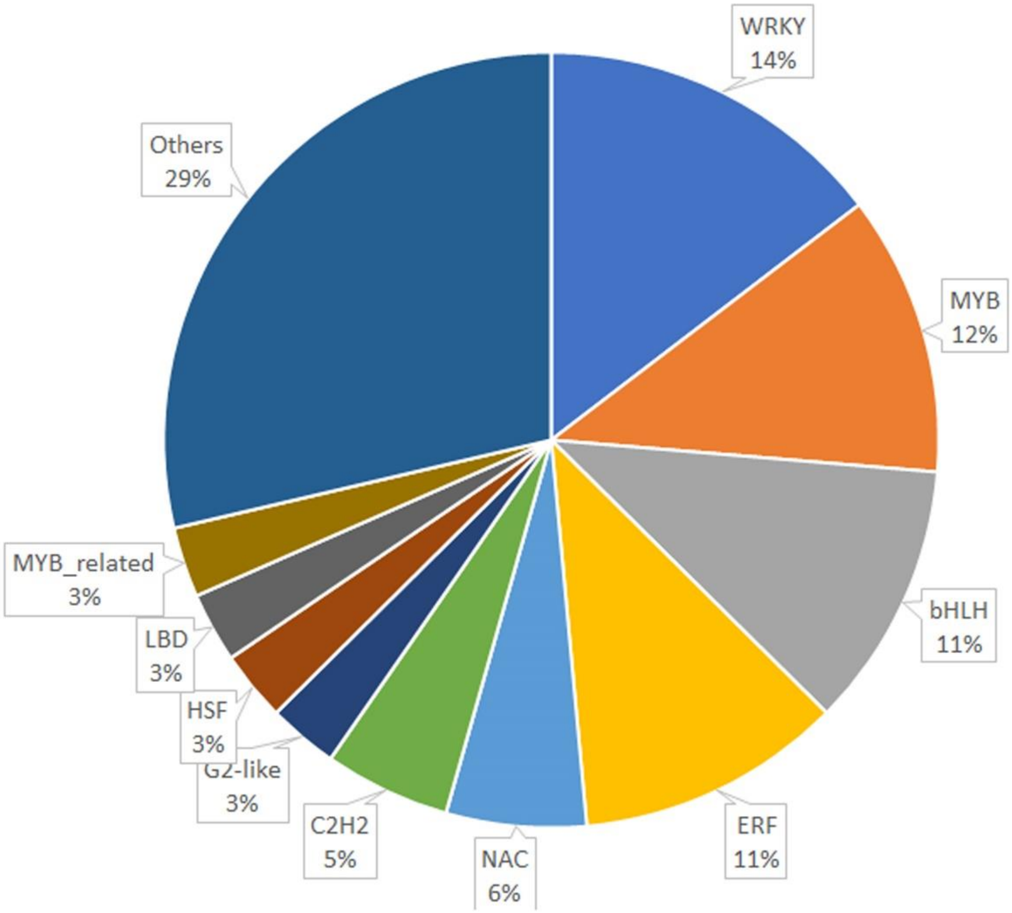

Figure S4 Proportion of the number of differentially expressed transcription factors of each transcription family out of the total differentially expressed transcription factors.
